# “If he could speak, he would be able to point out who does those things to him”: Experiences of violence and access to child protection among children with disabilities in Uganda and Malawi

**DOI:** 10.1371/journal.pone.0183736

**Published:** 2017-09-19

**Authors:** Lena Morgon Banks, Susan A. Kelly, Nambusi Kyegombe, Hannah Kuper, Karen Devries

**Affiliations:** 1 International Centre for Evidence in Disability, Department of Clinical Research, London School of Hygiene & Tropical Medicine, London, United Kingdom; 2 Gender Violence and Health Centre, Department of Global Health and Development, London School of Hygiene &Tropical Medicine, London, United Kingdom; Universita degli Studi di Perugia, ITALY

## Abstract

**Introduction:**

There is growing evidence that children with disabilities face an increased risk of violence globally. While child protection mechanisms to prevent and respond to violence–including formal government systems and more informal programmes and activities run by local communities or NGOs–are slowly becoming operationalised in low- and- middle-income countries, little is known about whether existing mechanisms are disability-inclusive. The aim of this study is to provide a better understanding of children with disabilities’ experiences of violence and their access to available child protection mechanisms in low resource settings.

**Methods:**

This study was conducted in Kasungu and Mulanje districts in Malawi and Kamuli district in Uganda between October-December 2015. In-depth, semi-structured interviews were conducted with approximately 20 purposively selected child/caregiver pairs in each country (43 pairs total). Interviews with key informants involved in the provision of child protection and disability support were also conducted. All interviews were recorded, transcribed and coded in NVivo. Thematic Analysis, complemented by constant comparison as described in Grounded Theory, was used to analyse the data.

**Results:**

Almost all **c**hildren with disabilities reported experiencing violence, with verbal abuse and bullying the most common forms. Very few of these children sought recourse through available child protection mechanisms. Some of the key factors impeding access to child protection for children with disabilities included: lack of local government disability-inclusive planning and budgeting; centralization of limited disability and social protection services; financial barriers to seeking and receiving care; and stigma and negative attitudes toward disabilities.

**Conclusion:**

Children with disabilities face both high levels of violence and high barriers to accessing available child protection mechanisms. There is an urgent need to ensure that all efforts to prevent and respond to violence against children are more disability-inclusive. In addition, it may be appropriate to target child protection mechanisms specifically toward children with disabilities because of the different and intersecting vulnerabilities that they face.

## Introduction

Every year, approximately one billion children around the world experience violence [1]. The World Health Organization defines violence against children as “all forms of physical and emotional ill-treatment, sexual abuse, neglect, and exploitation that results in actual or potential harm to the child’s health, development or dignity” [2]. Violence is a violation of children’s rights as enshrined under international laws including the United Nations Convention on the Rights of the Child (UNCRC), the African Charter on the Rights and Welfare of the Child (ACRWC), and the United Nations Convention on the Rights of Persons with Disabilities (UNCRPD). It is also a serious public health concern and a known risk factor for poor educational outcomes; externalising and conduct disorders; anxiety and depression; risky sexual behaviour; delinquency and criminal behaviour; negative interpersonal conflict resolution; drug and alcohol misuse; poorer health status in adulthood; and, increased risk of victimisation (for girls) and perpetration (for boys) of interpersonal violence in later life [3–14].

### Violence against children with disabilities

Some groups of children are known to be at particular risk of violence, including children with disabilities [15–19]. It is estimated that 150 million children are living with a disability, most of whom reside in low- and middle-income countries (LMICs) [20]. Children with disabilities are often amongst the most socially excluded and vulnerable [21], with a lower likelihood of attending school, a higher likelihood of experiencing serious illness, and a higher likelihood of living in poverty [22, 23]. Children with disabilities also appear more at risk of experiencing violence than their peers without disabilities [15, 16]. A recent systematic review of 17 studies from high income countries showed that one in four children with disabilities reported experiencing violence, of whom 20.4% reported physical violence and 13.7% sexual violence [16]. Overall, children with disabilities were three to four times more likely to be victims of violence than their peers without disabilities.

Few studies have investigated why children with disabilities are more at risk of violence. Possible reasons include lack of adequate support from carers, lower physical and emotional defences, communication barriers limiting defence from and reporting of violence, and a greater likelihood of being in vulnerable situations including being left in the care of non-related carers [16, 24]. Stigma and discrimination of disability is also a major contributor.

### Inclusion of children with disabilities in child protection mechanisms

Child protection mechanisms comprise the range of laws, policies, services, and activities dedicated to preventing and responding to violence against children [20]. Preventative actions include reducing social exclusion, providing support to families, and promoting safe communities [25]. On the response side, identifying cases of violence and its perpetrators (through community vigilance, policing, monitoring and reporting systems), determining appropriate steps to stop the perpetuation of violence (including access to justice), as well as victim support services (such as counselling and access to medical care) are all key.

Child protection mechanisms can be part of more formal, government-run systems, including policing, social welfare, and justice systems. However, more informal, community-based programmes and activities are also common, particularly in settings with weaker formal child protection systems. These can include local justice systems run by traditional leaders, volunteer child protection groups, community policing groups, parent support groups, or school-based activities. Actors involved in the provision of child protection can include the state, schools, community groups, children themselves, and increasingly in LMICs, non-governmental organisations (NGOs) [26].

Evidence on how to effectively reduce and respond to violence perpetrated against children with disabilities is also limited, particularly in LMICs. A systematic review identified 10 studies assessing the effectiveness of interventions to prevent and respond to violence against persons with disabilities; however, all but one study was conducted in a high-income country, and only two included children [26].

Children with disabilities may face particular difficulties in accessing child protection mechanisms compared to their peers without disabilities. For example, children with disabilities may face physical access barriers due to physical or visual impairments, social barriers arising from stigma and cultural beliefs about disability, and institutional barriers when existing mechanisms are not adapted to be inclusive of people with different types of impairments [27, 28]. However, there is a dearth of evidence in this area. Few studies have explored either experiences of violence or access to child protection mechanisms among children with disabilities in any context, but particularly in LMIC settings. Of the limited studies in this area, almost all focus on the perspectives of service providers, with little input from families and children with disabilities themselves. Due to the lack of attention to this topic, little consideration has been given to the inclusion of children with disabilities in child protection mechanisms at any level.

The aim of this paper is to provide a better understanding of the context of violence experienced by children with disabilities and if and how they access child protection mechanisms in Uganda and Malawi.

## Methods

Study methods were designed and reported in line with the Consolidated Criteria for Reporting Qualitative Research (COREQ) Checklist [29].

### Study context

This study was conducted in Kasungu and Mulanje districts in Malawi and Kamuli district in Uganda. These districts were selected as they are areas with some civil society provision of child protection, including activities supported by the project funder Plan International. These districts are predominately rural, with subsistence farming and small-scale agriculture and trading serving as the predominant livelihood activities. Poverty levels are high in all study locations. In Malawi, one third (Kasungu) and two-thirds (Mulanje) of the population lives in poverty (defined in national guidelines as having an annual per capita consumption below 37,000 MWK, equivalent to US$50) [30]. Food insecurity is also a major concern, affecting between 40–50% of the population in these districts [30]. In Uganda, more than 70% of those living in rural areas, such as Kamuli District, live in absolute poverty (defined nationally as less than US$1/day) or are classified as insecure non-poor (US$1-3/day) [31].

Recent data from Uganda reveal very high levels of violence among children attending primary school, with even higher levels among children with disabilities. In particular, girls with disabilities were found to be at significantly higher risk of violence when compared to their peers without disabilities [15]. Data from a 2013 national survey on violence against children in Malawi also indicates that there are high levels of violence against children in Malawi. Half of all girls and two thirds of boys reported experiencing physical violence, one in five girls and one in seven boys reported being sexually abused, and a quarter of children reported experiencing emotional violence [32]. Data were not, however, disaggregated by disability, making it impossible to determine the level of violence affecting children with disabilities.

Both Malawi and Uganda have ratified various international and regional regulatory frameworks and conventions addressing the rights and welfare of children, including children with disabilities (e.g. UN Convention of the Child, UN Convention of the Rights of Persons with Disabilities). In addition, both nations have passed national laws and policies regarding the rights and safety of all children, and have specific national level legislation in place specifically addressing the rights and needs of people with disabilities. In Uganda this includes The Children’s Act (1996 and Amendment 2015), National Policy on Disability (2006), and the Persons with Disabilities Act (2006) and in Malawi the Malawi Child Care, Protection and Justice Act (2010), and the Disability Act (2012). These national level policies outline the rights granted to all children, including children with disabilities, and the roles and responsibilities of the people and institutions tasked with their care.

### Sampling

In order to reflect a variety of participant characteristics, a purposive sample of at least 20 child-caregiver pairs was selected in each country. Children were selected based upon impairment type or condition, age (between 6 and 18 years old), gender, and school status (in-school and out-of-school). Children were identified through community-level key informants, for example through consultation with village heads, Disabled Peoples’ Organisations (DPOs) or through Plan International Malawi and Uganda district offices. In addition, key informants were selected and interviewed after consultation with the Plan International country and district level offices to ensure that a full range of relevant stakeholders were included. Invitations to participate were made face-to-face, mostly a few days before the interview date.

No respondents in Malawi or Uganda declined to participate in this study.

### Data collection

Data collection took place between October-December 2015. Children and caregivers were interviewed separately using semi-structured interview guides. Caregivers were invited to join the child interview if a child was unable to communicate independently (e.g. due to hearing impairment) or requested the presence of a caregiver. Interviews were conducted in the local languages (Chichewa in Malawi and Lusoga or Luganda in Uganda). Interview location was based on the interviewee’s preference, which for the vast majority was at their home. Privacy during interviews was enforced as much as possible so that interviewees could speak freely.

All interviews were audio recorded and were subsequently translated directly from local languages to English in a one-step process. For caregiver interviews, a semi-structured interview guide was developed to cover the following key topics in caregiver interviews: 1) background of the household and the child’s impairment; 2) understanding of what constitutes violence towards children; 3) caregiver safety concerns for their children and how their child’s impairment may or may not impact their risk of experiencing violence; 4) knowledge of and views on available child protection mechanisms; and 5) any experiences of violence and accessing child protection.

A semi-structured interview guide supported by visual aids was used during discussions with children. Storyboards with contextually relevant images of home, school and community were used while talking with children about the people, activities and experiences they encounter in each location. Children were asked about what makes them feel happy, sad, angry or afraid/unsafe in each context. Cards depicting children’s faces with these different emotional expressions were used with younger children to make the experience more participatory and as a communication aid for children with certain impairments. Children were also asked to whom and where they would go for help if they felt unsafe. They were then asked about whether they had ever experienced any type of violence (in line with the World Health Organization definition [2], including physical, sexual, and emotional abuse as well as neglect and exploitation). If they had experienced violence, they were asked whether they had sought help and what the response was like.

In all cases, information about the communication abilities of the children was sought in advance of the interviews in order to allow for adaptation or simplification of the questions if necessary. Interviews with children with intellectual or hearing impairments (with no formal sign language knowledge) were often more limited in scope, since effective communication with these children was limited; therefore, the ability to explore issues in more depth was constrained. Sign language interpretation was available; however, none of the children in the sample had knowledge of formal sign language. In some of these cases, other individuals close to the child, such as a sibling, relative, or friend was consulted in order to gather additional information.

Key informant interview questions were tailored to each individual’s area of expertise but broadly focused on: risks of violence for children with and without disabilities; available child protection mechanisms; and, any barriers or enablers that children with disabilities may face in accessing these mechanisms. Most key informant interviews were conducted in English and typically took place at the interviewee’s workplace unless other arrangements were requested.

### Research team and reflexivity

Interviewers were Ugandan and Malawian women who did not know the study participants and were not affiliated with Plan International. Interviewers were mostly university-educated and had worked previously in research, including with qualitative methods and with children with disabilities. A two-day training of the interviewers was undertaken by the lead London School of Hygiene & Tropical Medicine (LSHTM) field researchers [LMB, SAK], which included modules on children and violence, disability, child protection, ethics of conducting research with children, study background and methods, interview techniques as well as referral pathways in the event of disclosures of violence. Initial interviews were guided by LSHTM lead field researchers, with interviewers progressively leading the discussions independently. While the LSHTM lead field researchers accompanied the research team during data collection, they typically maintained a distance from the interview site, as it was felt that the presence of additional people, particularly foreigners, could disrupt the privacy and rapport between the interviewer and interviewee.

To reduce interviewer bias, improve competency, and identify gaps in topics covered, early-stage interview transcripts were reviewed by the lead LSHTM field researchers and discussed with interviewers.

### Analysis

Thematic Analysis, complemented by constant comparison as described in Grounded Theory, was used to analyse findings [33]. After each day of fieldwork, field debrief sessions were held and the interview notes were reviewed by the lead field researcher and the interviewers. These immediate feedback discussions helped to identify gaps in the interview schedule, clarify interview questions or issues that needed additional probing, identify emergent themes, and provide alternative points of view to minimise researcher bias.

An initial coding framework based on review of the literature, previously conducted research, and the structure of the interview tools was developed and adapted to support the analytical needs and themes identified within the data collected from each country. All the transcripts were read prior to coding to identify additional themes and codes to add to the framework. Data were coded and analysed for each country separately using NVivo 10 (by LMB for Malawi and SAK for Uganda with input from NK). After country-specific analyses was completed by the lead LSHTM field researchers, findings between Uganda and Malawi were compared before being combined at the point of interpretation. In-country partners in Malawi and Uganda provided feedback on the final themes.

### Ethical considerations

Before the start of each interview, informed written consent was obtained from key informants, caregivers, and older children (above 16 years). For younger children and children with communication or intellectual impairments a simplified oral assent was sought in addition to caregiver consent. In this study, disclosures of severe physical violence or sexual violence against a child under the age of consent warranted a mandatory referral to relevant health and child protection services; for other less urgent child protection concerns or for children above the age of consent, an optional referral was offered. Participants were made aware during the consenting process that certain disclosures of violence would necessitate this breaking of confidentiality. This protocol has been used in other studies exploring violence towards children with disabilities [15]. Similarly, if children with disabilities had unmet health or rehabilitation needs, referrals to nearby services were offered. Plan International country and district offices were notified and responsible for all follow-up and provision of additional support.

Ethical approval for this study was obtained from the recognised ethics committees in both Malawi and Uganda, as well as the ethical committee of the lead research institution.

## Results

### Description of the study sample

Key characteristics of the study samples in Malawi and Uganda are summarized in Table 1.

**Table 1:** Characteristics of study sample.

	Malawi	Uganda
**Average age in years (range)(range)**	13 (6–18 years)	12 (6–18 years)
	**# children in sample**	
**Gender**	10	10
Girls	12	11
Boys		
**Impairment type/condition**		
Physical	9	3
Intellectual	4	1
Hearing	4	4
Visual	4	1
Epilepsy	3	5
Albinism	2	0
Multiple Impairments	7	7
**School status**		
In	13	6
Out of school	8	15
- Dropped out	7	9
- Never enrolled	1	6

In total, information on 22 children was gathered through 21 caregivers and 17 child interviews in Malawi and on 21 children through 29 caregivers and 13 child interviews in Uganda (see Table 1). Out of the 43 child-caregiver pairs, five children in Malawi and eight in Uganda where not directly interviewed due to communication difficulties related to their impairments (e.g. intellectual impairments and/or profound hearing loss without knowledge of formal sign language). Although attempts were made to communicate with these children through other means (e.g. use of visual tools, involving household members for interpretation using homemade sign language), information gathered through these avenues was limited.

Twelve key informant interviews took place in Uganda and eighteen key informant interviews and one focus group discussion in Malawi. Key informants included members of Disabled People’s Organizations (DPOs) and NGOs, rehabilitation and disability support providers, teachers, formal district- and national-level child protection service providers (e.g. police, justice system and social welfare officers), and community-level child protection providers (e.g. volunteer policing groups, support groups, and local leaders).

### Context and experiences of violence

#### Perceptions of violence

When caregivers were asked what they believed constituted violence towards children, forms of physical and sexual violence and neglect were frequently cited. While sexual violence was always considered to be abuse and viewed as affecting primarily girls after puberty, physical violence was seen on more of a spectrum. For example, corporal punishment by adult family members or teachers, though still seen as undesirable and even cited as a form of abuse by many caregivers during interviews, was still reported to be commonplace and generally accepted as a form of discipline: “The common form of violence here is whipping a child… if she/he does something wrong, for instance playing with the water that you have worked hard drawing under the sun…[still] they [parents] regret after they have already whipped the child.” [Malawi, caregiver]. Indeed, most children interviewed reported experiencing corporal punishment.

Similarly, forms of child exploitation, neglect, and verbal abuse were also viewed fluidly. Commonly cited forms of violence included “not providing food or clothes”, “shouting unnecessarily”, and “making children…carry heavy things when you know that she can’t manage”. Interestingly, denying children an education and, in Malawi, child marriage were often perceived as forms of abuse by caregivers, which may reflect recent awareness campaigns by NGOs in their communities. Still, many caregivers acknowledged difficulties in safeguarding their children from all forms of perceived violence given the realities of their daily lives. For example, some caregivers admitted difficulties minding their children due to heavy workloads or asking their children (with or without disabilities) to miss school in order to work; though they perceived this situation as undesirable, it was also seen as unavoidable, given the high levels of poverty they experienced.

#### Experiences of violence

The majority of the children with disabilities in both the Malawi and Uganda samples reported experiencing some form of violence (Malawi n = 20/22; Uganda n = 13/21). Children interviewed reported experiencing physical and emotional abuse (e.g. bullying, abusive name calling), as well as stigma and isolation. Children with disabilities reported that peers were common perpetrators of violence, although there were also reports of violence perpetrated by caregivers, family members, teachers, and other adult community members. Many children and caregivers suggested that the violence and abuse the children experienced was directly related to their disability for a variety of reasons.

Both caregivers and key informants in Malawi and Uganda reported neglect and social isolation as forms of violence frequently experienced by children with disabilities. Key informants suggested that children with disabilities are believed to be a curse bestowed on their mothers or family due to past or present transgressions. In addition to negative associations with witchcraft and the supernatural, these children may be seen as economic and social burdens to families and communities, which can lead to neglect, abandonment, and social isolation. For example, one boy in Malawi explained that his mother “says I shouldn’t be staying with her…. [when I get close to her house] she talks about my leg… and swears at me [to go away].” Consequently, he stays with his grandmother, even though his mother lives nearby. Other cases of parental abandonment, often linked to the stigma of having a child with a disability, were also reported.

Even without overt discrimination and neglect, caregivers of children with high support needs highlighted the challenges of providing the needed level of care and supervision to keep their child safe at all times. Many caregivers reported diverting large amounts of time away from livelihood activities in order to care for their child:

“I had a difficult time leaving him alone at home because during those times he was having convulsions the whole day…. I was feeling sad and even failed to cook for my family…. I couldn’t do household chores because I couldn’t afford leaving him lying down [in a convulsive fit].” [Malawi, caregiver]

Sexual violence against children with disabilities—especially girls—was expressed as a major concern of caregivers and key informants. In Uganda, caregivers of two girls with disabilities reported that their girls had experienced sexual abuse, while a staff member from an NGO working in the field of child protection explained:

“…Yeah they take advantage. For one, if you can’t talk, someone [can] just grab you and take you to the bush. They defile them because they can’t make any noise. They can’t speak. They can’t say no. They can’t raise an alarm. So, it puts them under a very dangerous circumstance. Though we are saying children are children… they are all minors, but they [children with disabilities] are more vulnerable when we do that assessment. They are more vulnerable than someone who can speak, walk, run… Like that girl you found in the wheelchair, someone can take advantage…. how will you crawl very fast? So, they will use that advantage to do what they want.” [Uganda, NGO staff member]

### Preventing and responding to violence: Access to child protection mechanisms

A mix of child protection mechanisms to prevent and respond to violence were available in Malawi and Uganda. These included informal or community-based activities supported by NGOs and local community leadership bodies; for example, support groups for parents of children with disabilities, volunteer child protection committees, community policing, and sensitization activities. More formal government-run services such as police, social welfare, and bodies of the justice system were also available although predominately at district centres.

Awareness of child protection mechanisms, such as formal or informal policing or various community-based activities was high, particularly among caregivers; however, awareness was lower among children–particularly younger children and children with impairments that affected communication. For both caregivers and children, detailed knowledge on where to go to access each mechanism and what services they could provide was limited.

Despite the high levels of violence reported by caregivers and children with disabilities in both Uganda and Malawi, few had accessed child protection mechanisms. The factors that impacted children’s access to child protection mechanisms, including preventative actions to reduce their risk of violence, are outlined below.

### Lack of disability-inclusive programming in child protection

The vast majority of key informants, as well as some caregivers in both countries, cited the lack of resources for child protection as a primary barrier to the provision of services. Some shortfalls in the availability of key inputs, such as dedicated child protection budgets and skilled professionals working in child protection, affect all children. However, children with disabilities face additional challenges arising from the lack of disability-inclusive programming. Often, existing mechanisms are not adapted to accommodate the needs of children with different impairment types (e.g. built environments that are inaccessible for children with physical and visual impairments, lack of alternative forms of communication for children with hearing or intellectual impairments). Additionally, few disability-specific initiatives are in place to address the particular vulnerabilities faced by children with disabilities, such as tackling stigma and discrimination and providing assistance to caregivers of children with high support needs.

While both countries have enacted legislation within the last decade which promotes disability-inclusivity and universal access, including in the delivery of key services such as child protection, gaps in implementation remain. For example, as a DPO member in Uganda explained: “the accessibility standards are by law that all public places are supposed to be accessible… [but if] a child who is moving in a wheelchair goes to the police to report a case, it will end there. [The child] will not reach to the offices. The place is not accessible.”

In both Uganda and Malawi, children with impairments that affected their ability to communicate–such as intellectual impairments or profound hearing impairments–appeared particularly at risk of violence and faced additional barriers to accessing existing child protection mechanisms. Most importantly, for children who had experienced violence, difficulties sharing what had happened to them, including identifying the perpetrator, could lead to continuing abuse:

“People beat him up and sometimes he comes back home crying and with bruises on his face… [and] his body swollen from the beatings. He goes straight in bed and cries himself to sleep…. It worries me and sometimes I feel like crying because my child goes through that. If he was able to speak, he would be able to point out who does those things to him.” [Malawi, caregiver]

Almost none of the child protection providers interviewed had strategies in place or resources available for working with children with communication impairments (e.g. simplified text, audio-visual formats, sign language interpretation). This lack of training and resources–combined with the time pressure from high workloads–was reported to lead to the neglect of cases involving children with communication impairments. For example, in Malawi, police recounted how difficulties gathering evidence due to communication challenges often led to delays or failures in resolving cases involving children with disabilities. Even in the rare cases when specialist resources such as formal sign language interpretation are made available, children cannot benefit if they have not been trained in these methods of communication themselves. This was evident in the interviews conducted for this study, as none of the children with profound hearing impairments were trained in formal sign language, underscoring poor access to disability-inclusive education.

### Geographic accessibility

Formal child protection services, such as police, branches of the justice system, and social welfare offices, as well as healthcare and counselling services are predominantly urban-based in both Malawi and Uganda. Consequently, when the involvement of these more formal services is required, many key informants and caregivers in both countries cited geographic accessibility as a challenge for all children, with and without disabilities. However, the long distances, difficult terrain, lack of accessible transportation, and need for accompaniment–combined with building access issues upon arrival–appeared to pose a particular problem for children with disabilities. The magnitude of the challenges faced in simply getting to the relevant services could discourage families from reporting violence or continuing to pursue resolution to their case, particularly for complex cases requiring long-term follow-up. The father of an 8-year old girl with profound hearing, speech and intellectual impairments talked about how he did not to pursue further assistance for his daughter after an attempted rape because he could not afford the time and money required to travel over 150km each way to the specialised services available in the capital.

In both Uganda and Malawi, informal or community-based child protection mechanisms have provided decentralised avenues for addressing some child protection concerns. However, physical barriers still limited access to even these mechanisms, particularly for children with mobility impairments. As many children with mobility impairments had not received assistive devices or rehabilitation services, they were often dependent on others to facilitate access. As one boy in Malawi with a physical impairment and no assistive device explained when asked where he would go for help if he experienced violence, “I can’t do anything because I can’t walk…I can’t tell anyone else…I can’t crawl to far distances.”

### Financial vulnerability

Nearly all caregivers and key informants pointed to financial barriers as a major deterrent to both seeking and providing needed child protection mechanisms. While most mechanisms are designed to be free, even indirect costs, such as for transport and missed time from work, were perceived as sufficiently onerous for many families to de-incentivize seeking services.

Additionally, particularly in Malawi, it was widely reported that it was common practice for police, traditional leaders, and other child protection groups to demand payment in return for following up on their case—even when these activities are supposed to be free. Some service providers confirmed this practice by explaining that severe shortages and unpredictable flows in funding necessitated the unsanctioned demands for payment, as without them, they could not finance even the most basic activities such as transportation to follow-up on cases or paper to log and track cases. Caregivers and other key informants not involved in service provision felt, however, that corruption may be more at the heart of this practice. Some reported incidents where child protection bodies “receive money from the perpetrators and then the case just dies down” [Malawi, caregiver]. In either case, many caregivers felt that money played an instrumental role in determining who was able to access child protection, with the view that “they [police and other child protection bodies] favour families that are economically well and delay assisting poor families…. it starts from these [community-based] groups [and goes] up to police.” [Malawi, caregiver]

Furthermore, financial vulnerability can increase risk of violence and dissuade families from responding. As explained by a father in Uganda whose daughter experienced an attempted rape: “if [a] perpetrator is offering a million [less than $300 USD] to a broke or poor family for defilement, they just agree and let their daughter suffer. So, that means everyone with a million [Ugandan Shillings] can commit that crime and the child doesn’t receive any justice.”

While the study areas in both Uganda and Malawi all experienced high levels of poverty, households with children with disabilities may face particularly extreme deprivation, exacerbating vulnerability to violence and the financial accessibility of services. Many households reported spending on costs related to their child’s disability–for example, paying for transportation to reach distant hospitals or purchase medications and assistive devices–as well as diverting time away from work to care for their child, accompany them to school or on frequent healthcare visits as reducing households’ already constrained resources. Additionally, in both Uganda and Malawi, over half of the children in the study were not living with both their parents, with several reporting parental abandonment. Consequently, many children with disabilities not only lived in households with fewer adults able to provide the needed level of care and supervision, but also in households with fewer economic providers, particularly given that mothers or grandparents were most often the remaining caregiver.

### Stigma and discrimination

Discrimination and stigmatising cultural beliefs are potentially both a cause of increased violence towards children with disabilities and a barrier to accessing child protection. Key informants and some caregivers noted that discrimination and negative attitudes–by service providers, family and the community alike–can normalise violence towards children with disabilities. For example, children with disabilities can be seen as burdens with little ability to contribute to household and community wellbeing. One caregiver in Uganda explained: “…the parents have rejected these children with disability because they say that they are good as nothing… there is nothing good in them and the other issue is that most people know that the sickness they suffer from is incurable. So that’s why they are being left out.”

These negative attitudes toward disability could lead to increased vulnerability to violence. Furthermore, it could lead to cases not being reported, being de-prioritised by child protection bodies, or being responded to insufficiently with lighter punishments given to perpetrators than would be typical for similar cases involving children without disabilities. One DPO member in Malawi reflected on a domestic violence case where a child’s stepfather “hit the child so hard that he was bleeding” but was not punished according to what he perceived would be typical standards because “maybe they [police] gave him that mild punishment because the child had a disability…they would’ve given a much stronger punishment if it involved a child without disability.”

### Acceptability around the use of child protection mechanisms

Norms around the role of children in society can prevent children from accessing services independently without the involvement of an adult. When asking children where they would go if they were to experience different forms of violence, almost all said they would go to their parents or another close adult contact. Even if they were aware of other child protection mechanisms, most children in Malawi reported that they would need to go through an adult to access them, noting for example, that a chief “would look down on me and not listen to what I have to say.” As key informants noted that close contacts could be the perpetrators of violence, children may not be able to report their experiences unless there are avenues available that they can access autonomously.

Girls with disabilities may face additional difficulties independently accessing child protection mechanisms due to norms around gender. For example, in Malawi, several female caregivers indicated that if their child were to experience violence, it would be their husband or another male relative who would report the abuse rather than either themselves or their daughters.

Finally, attitudes on the acceptability of when and where to seek recourse for abuses against children could lead to the continued propagation of violence towards children with disabilities. For example, although bullying by peers and verbal abuse–most of which was believed to be disability-targeted–was the main type of violence experienced by children with disabilities and was cited as a major concern, it was largely seen as inevitable. Although some caregivers sought to resolve this type of violence by speaking with the perpetrator, the perpetrators’ family, or school staff, many caregivers reported that their efforts had been unsuccessful at stopping the abuse and they had not sought further recourse. Furthermore, most children and caregivers alike indicated a preference to resolve issues within the community, noting that there could be repercussions to community cohesion and their relationships with others if they sought the involvement of more formal child protection actors.

## Discussion

This study aimed to explore children with disabilities’ experiences of violence and accessing child protection mechanisms in Uganda and Malawi. Our key finding is that while children with disabilities experience high levels of violence, access to available child protection mechanisms is lagging. Some of the key factors impeding access to child protection for children with disabilities included: lack of disability-inclusive planning and budgeting—particularly to promote the inclusion of children with communication impairments; centralization and poor physical accessibility to the limited available services; financial vulnerability; and, attitudes around disability and the acceptability of when and where to seek recourse to violence.

A small number of studies and policy reports on this topic corroborate some of our findings. For example, the long distances to centrally delivered programmes and services, combined with the lack of accessible transportation and inaccessible facilities have previously been highlighted as key challenges, particularly for children with mobility impairments [27, 28, 34–36]. Lack of disability-inclusive planning and budgeting, such as training of professionals on how to work with children with disabilities and provision of accommodations (e.g. alternative forms of communication such as Braille, sign language), has similarly been noted [27, 28, 34, 35, 37]. Furthermore, there is increasing awareness that children with communication impairments appear both more susceptible to violence and less likely to access children protection mechanisms [27, 28, 34–36, 38]. Finally, discrimination among service providers and lack of awareness of available services among children with disabilities have also been documented [28, 37].

Almost all of these studies and policy reports have focused on the opinions and experience of stakeholders working in child protection. However, there is a dearth of research exploring the perspectives of children with disabilities and caregivers themselves, especially in resource-limited settings. A key strength of this research is that by focusing on these often-overlooked viewpoints, a more nuanced understanding emerges of how overlapping layers of vulnerability can combine to exacerbate risk of violence and barriers to accessing child protection. Globally, and particularly in low resource settings, violence against all children–with and without disabilities–is very high and child protection mechanisms are often inadequate to meet many children’s needs [1, 32]. While some challenges are universal to all children–for example, low availability of child protection resources, financial and geographic barriers and the acceptability of autonomous access for children–this research indicates that children with disabilities may face either additional or heightened restrictions.

For example, the relationship between disability and poverty can help explain why children with disabilities may be more at risk of violence and face heightened barriers to accessing child protection mechanisms. There is strong evidence globally that households with members with disabilities are significantly more likely to live in poverty [23]. While poverty was high in the study regions, narrative from the children with disabilities and their families in the sample indicated that they may be experiencing even greater levels of deprivation than average. For instance, many caregivers reported diverting large amounts of time away from livelihood-supporting activities for caretaking responsibilities and spending on costs related to their child’s disability. This phenomenon of “extra costs of disability” is increasingly recognised as a major contributor to poverty amongst households with a member with a disability [39–41]. Furthermore, in both Uganda and Malawi, over half the children were not living with both parents, often in households headed by their mother or grandmother. Not only did this limit the number of economic providers, but caregivers, particularly of children with high support needs, faced difficult decisions between balancing time spent ensuring their child had the needed level of care and supervision and time devoted to livelihoods. In several cases, caregivers reported that they were unable to provide sufficient supervision to ensure their child’s safety at all times. These caregivers perceived that this increased their children’s risk of experiencing violence and was a barrier to accessing existing mechanisms.

Similarly, access to education as well as health and rehabilitation services are important mitigating factors that can potentially prevent violence and improve access to child protection, yet are not always available for children with disabilities [22]. Schools can provide supervision as well as avenues for accessing child protection mechanisms independent from their caregivers; exclusion from education can contribute towards increased isolation and vulnerability. Furthermore, none of the children with communication impairments in the sample had received specialised training in sign language and other forms of alternative communication, thereby limiting their ability to disclose experiences of violence and advocate for themselves. However, even when children with disabilities do attend school, they may still experience violence and social exclusion while at school if these institutions are not themselves disability-inclusive and if discriminatory attitudes about disability prevail among peers and teachers [42]. Meanwhile, on the side of health and rehabilitation, access to these services—including the provision of assistive devices—can greatly increase children’s level of functioning and independence.

Through the findings of this research, it is clear that a holistic approach is needed to both prevent and respond to violence against children with disabilities considering the different and intersecting vulnerabilities that these children face. In considering interventions, there is a need to adopt a “twin track” approach. In addition to adaptations to existing mainstream programmes and services to promote equal access for children with disabilities, some disability-specific interventions may be required. For example, to prevent violence, there is a need for increased sensitisation around disability, particularly in communities and in schools, to decrease negative attitudes around disability and combat the disability-targeted elements of abuse. Similarly, specific programmes to provide additional support to caregivers, decrease social exclusion of children with disabilities in areas such as health and education, and tackle the link between disability and poverty can help address some of the drivers of children vulnerability to violence and barriers to receiving recourse for children with disabilities.

At the same time, disability-inclusive planning and evaluation of existing child protection mechanisms are essential for promoting equal access amongst all children. There is a clear need to increase training of individuals engaged in the provision of child protection about disability, at all levels, including school- and community-based programmes as well as more formal services. While training of more formal service providers in disability is increasing, most trainings appear to focus exclusively on the principles of non-discrimination, but do little to equip individuals with a practical knowledge of–or budgets for–strategies on how to work with children with disabilities and provide needed accommodations.

More research is needed to evaluate existing or trial new interventions to identify elements of good practice in designing disability-inclusive child protection mechanisms for low resource settings. Evidence based policy is lacking, even in high income countries[26]. One recent intervention to address violence in schools in Uganda has shown promise in reducing violence against all children in schools, including children with disabilities [15]. More evaluation of existing interventions, disaggregated by disability, is essential for ensuring the benefits of these programmes are shared equally among all children. In addition to assessing disability-inclusion in these mainstream schemes, our research has highlighted a lack of disability-specific child protection interventions, indicating a need to design, trial, and assess these types of interventions as well.

Furthermore, while this qualitative research provides an in-depth exploration of factors which may increase the risk of violence among children with disabilities and limit their access to child protection mechanisms, quantitative research to assess the magnitude of these challenges is needed, particularly in comparison to child without disabilities. Furthermore, similar research in other countries and settings could further illustrate how these challenges vary by context.

### Limitations

In interpreting the findings of this research, some limitations should be taken into consideration. While efforts were made to capture the voices of all children with disabilities, the evidence gathered from interviews with children with communication impairments was more limited in nature. Although triangulation with other household members and friends elicited additional information about these children, some of the experiences of this group of children may not have been fully captured. Additionally, the study districts were all rural and marked by high levels of poverty, limiting generalizability to other settings. Most of the children in the sample could be classified as having moderate to severe impairments, so findings may not be as relevant for children with more mild impairments. Finally, few children who had experienced violence had actually sought recourse through more formal child protection mechanisms, making it difficult to clearly identify facilitators to receiving services.

## Conclusion

Exposure to violence in childhood can have wide-ranging social, physical, and emotional consequences across the life-course. While children with disabilities face a heightened risk of many forms of violence, they also appear to face additional barriers that limit their access to child protection mechanisms. There is an urgent need to ensure that all efforts to prevent and respond to violence against children are more disability-inclusive.
